# The intersection between sex and drugs: a cross-sectional study among the spouses of injection drug users in Chennai, India

**DOI:** 10.1186/1471-2458-11-39

**Published:** 2011-01-16

**Authors:** Sunil S Solomon, Aylur K Srikrishnan, David D Celentano, Sethulakshmi C Johnson, Canjeevaram K Vasudevan, Kailapuri G Murugavel, Santhanam Anand, M Suresh Kumar, Suniti Solomon, Shruti H Mehta

**Affiliations:** 1 YR Gaitonde Centre for AIDS Research and Education, VHS Adyar, Taramani, Chennai 600113, India; 2 Johns Hopkins Bloomberg School of Public Health, 615 N Wolfe Street, Baltimore, MD 21205, USA

## Abstract

**Background:**

It is estimated that there are up to 1.1 million injection drug users (IDUs) in India; the majority are likely married. We characterize HIV, hepatitis B (HBV) and hepatitis C (HCV) prevalence and the risk environment of a sample of spouses of IDUs.

**Methods:**

A cohort of 1158 IDUs (99% male) was recruited in Chennai, India from 2005-06. A convenience sample of 400 spouses of the male IDUs in this cohort was recruited in 2009. A risk assessment questionnaire was administered and a blood sample collected. Logistic regression was used to identify factors associated with prevalent HIV.

**Results:**

Median age was 31 years; thirteen percent were widowed and 7% were not currently living with their spouse. Only 4 (1%) reported ever injecting drugs; Twenty-two percent and 25% reported ever using non-injection drugs and alcohol, respectively. The majority had one lifetime sexual partner and 37 (9%) reporting exchanging sex. Only 7% always used condoms with their regular partner. HIV, HBV and HCV prevalence were 2.5%, 3.8% and 0.5%, respectively; among spouses of HIV+ IDUs (n = 78), HIV prevalence was 10.3%. The strongest predictor of HIV was spousal HIV status (OR: 17.9; p < 0.001). Fifty-six percent of women had ever experienced intimate partner violence; Eight-six percent reported sexual violence.

**Conclusions:**

Our finding of a 10-fold higher HIV prevalence among spouses of IDUs compared with general population women indicates their vulnerability; prevalence is likely to increase given the context of low condom use and frequent sexual violence. Prevention efforts directed at IDUs should also include programs for spouses.

## Background

Injection drug users (IDUs) are at high risk for HIV acquisition and currently drive several of the fastest growing HIV epidemics globally [1-4]. In India, the HIV epidemic has historically been driven by heterosexual transmission from initial reports among female sex workers (FSWs) to eventual documentation of high HIV prevalence among women who were married and monogamous and reported no risk factors for HIV other than sex with their husbands [5,6]. However, recent reports have suggested stabilization of the heterosexual epidemic with decreasing prevalence and incidence among FSWs and antenatal clinic attendees [7,8]. This is likely in part due to interventions (e.g., condom promotion, sexually transmitted infection [STI] testing) targeted over the past decade at heterosexual high-risk populations including FSWs and truck drivers.

Concurrently, HIV epidemics among other risk groups such as IDUs have continued to grow [6]. Though there has been a wide range in the estimated size of the IDU population in India due to use of different estimation techniques, it has been suggested that up to 1.1 million IDUs [1,4] may reside in India. In the early years of the epidemic, HIV among IDUs in India was concentrated in the Northeastern states (due to proximity to the 'Golden Triangle' - Burma, Laos, Thailand and Vietnam). However, it is now evident that IDUs exist in most major metropolitan cities in India and have high prevalence of HIV and associated blood-borne infections (e.g., hepatitis B virus [HBV] and hepatitis C virus [HCV]) [9-13]. Beyond transmission within drug using networks, IDUs can also transmit HIV and other blood-borne infections heterosexually because the majority are married and sexually active [14-16]. Transmission to wives and other sexual partners is likely facilitated by low rates of condom use with regular sex partners which has been previously reported among IDUs in India [17]. Thus, the wives of IDUs represent another group of married monogamous women who are at risk for HIV and other infections primarily because of their husband's high risk behavior [18-20].

Prior studies in India have characterized the prevalence of HIV among spouses of IDUs; a range of prevalence has been observed from 5% in Chennai to 45% of those in Manipur [14-16]. However, none of these studies characterized prevalence of other bloodborne infections including HBV and HCV. Further, few have characterized the context of risk for these women; understanding their own level of risk behavior, their economic situation and their relationships with their husbands will provide a broader understanding of their risk environment and will help to design programs for this potentially vulnerable group [21]. Of particular interest is experienced intimate partner violence (IPV). Violence against women is recognized as a global public health problem and can have adverse impacts on physical, mental and reproductive outcomes and can also increase risk for HIV [22-24]. A number of studies have demonstrated high levels of IPV in India [25-28], but few studies have characterized violence among spouses of IDUs. As violence has been associated with alcohol use [27], it is possible that experienced violence may be even greater in this population and can further influence risk for HIV and other infections.

We previously characterized the prevalence of HIV, HBV and HCV infection in a cohort of 1158 IDUs in Chennai, India to be 25%, 11% and 65%, respectively [29]. Almost two-thirds of the IDUs were married of whom 40% were also sexually active. The objective of this study was to characterize the prevalence of HIV, HBV and HCV infections among a sample of the wives of the IDUs in this cohort as well as to understand their risk environment.

## Methods

### Study Population

Between April 2005-May 2006, 1158 IDUs were recruited into a longitudinal cohort in Chennai, India [29,30]. The Madras Injection Drug Users and AIDS Cohort Study (MIDACS) operates through the YR Gaitonde Centre for Substance Abuse-Related Research (YRGCSAR) in north Chennai. YRGCSAR was established in November 2004 to provide HIV voluntary counseling and testing (VCT) services to marginalized populations and conduct longitudinal assessments of HIV incidence and drug abuse among IDUs in Chennai. A convenience sample of IDUs was recruited through extensive community outreach as previously described [29]. Briefly, field staff, who were predominantly former IDUs, recruited participants from locales in all zones of Chennai where IDUs were known to congregate (e.g., shooting galleries, drug treatment centers, etc.). Participants were eligible if they (1) provided written informed consent, (2) were at least 18 years of age, and (3) injected at least once in the prior 6 months by self-report. Seven hundred and forty-five (64%) of the 1158 participants were married.

Between June and August 2008, we conducted four focus groups among male IDUs (n = 38) and four focus groups among known spouses of male IDUs (n = 33) to determine the best methods to recruit spouses of IDUs into a research study. While greater than 75% of the male IDUs were interested in their wives participating in a study and receiving HIV testing, most were too afraid to bring them in themselves. IDUs and their spouses revealed that the majority of women married to IDUs were aware that their husbands were injectors but few were aware of their husband's HIV serostatus. Most IDUs wanted assistance in disclosing their HIV status to their wives. This study provided them with an opportunity for disclosure in a setting where couples counseling would be available. Based on this formative work, we adopted two methods of recruitment. IDUs in the ongoing MIDACS were told that they could bring their wives into the clinic for participation in the study. They were assured that disclosure of any personal information to their wives would only be done at their request and consent. In addition, IDUs provided study staff with contact information for their wives and these women were approached directly by YRGCSAR staff. Study staff have worked in the communities of North Chennai since 1999 and have excellent rapport in this region where the majority of MIDACS participants live. As the recruitment of wives took place nearly three years after enrollment of the IDU cohort and a high rate of mortality in this cohort (secondary to drug overdose, HIV and tuberculosis) has been observed [31], 13% of IDUs were dead by the time their wives were contacted. This study was approved by the YRGCARE and Johns Hopkins Bloomberg School of Public Health institutional review boards.

The recruitment for the cross-sectional study took place between April-November 2009. A convenience sample of predetermined size (n = 400 women) was recruited (representing 54% of the wives of all married IDUs in the MIDACS). Women were eligible to participate if they were ≥18 years of age, provided written informed consent and reported having a husband who had a history of injection drug use. To achieve the sample size of 400, 433 women were screened; thirty-three (7.6%) did not consent. As all women were known spouses of IDUs in the ongoing MIDACS, consent was also obtained to link their HIV, HCV and HBV results with those of their husbands who were enrolled in the MIDACS. Even women who were recruited independently provided identification information that permitted linking their laboratory data with their spouses who were enrolled in MIDACS. However, behavioral data from the MIDACS was not linked with the behavioral data from this study as per the mandate of the IRB.

### Data collection

A blood sample was collected to test participants for HIV, HBV and HCV and a risk assessment questionnaire was administered to all participants (additional file 1). HIV status was ascertained by testing for antibodies to HIV by duplicate ELISA (Murex HIV-1.2.O, Abbott Murex, UK and Vironostika^® ^HIV Uni-form II Ag/Ab, bioMérieux, The Netherlands). Antibodies to HCV (anti-HCV) were identified using the Murex Anti-HCV kit (Abbott Murex, Republic of South Africa), and chronic hepatitis B virus (HBV) infection was diagnosed by the presence of hepatitis B surface antigen (HBsAg) (Hepanostika HBsAg Uniform II, Biomérieux, The Netherlands). All participants were provided with pre-test counseling prior to collection of the blood specimen. Additionally, participants were asked to return to YRGCSAR two weeks later for results, post-test counseling and referrals for HIV care, if needed. The risk assessment questionnaire collected information on demographics, history of substance use (alcohol, non-injection and injection drug use), sexual risk behavior (including main and non-spousal partners, condom use and commercial sexual encounters), intimate partner violence (IPV) and perceived impact of drug use on the family unit. Women were asked about their lifetime experience with verbal, physical and sexual violence; they were also asked to document the number of episodes of each in the prior six months. They were asked about knowledge of their spouses drug use, HIV, HCV and HBV status. Finally, they were asked to disclose all of the ways in which their husband's injection drug use impacted their family (e.g., economic deprivation, negative influence on children, violence) and then to state their primary concern about their husband's drug injection.

### Statistical Analysis

Analyses presented are primarily descriptive. Dichotomous/categorical variables are described as n (%) and continuous variables as median (interquartile range [IQR]). Univariate logistic regression was used to identify factors associated with prevalent HIV infection. As only 10 women were infected with HIV, multivariate analysis was not performed. All analyses were performed using Intercooled STATA Version 10.1 (College Station, Texas, USA).

## Results

### Description of population

The median age was 31 years (IQR: 26-36), the majority were of Tamil ethnicity (97%) and had either no (28%) or only primary-level education (62%). Eighty percent were married and living with their spouse, 7% were living away from their spouse and 13% were widowed (Table 1). The median duration of marriage was 14 years (IQR: 7-19). The majority were employed and earned daily wages. However, reported family income was very low; sixty-seven percent reported an income of less than INR 500 (10 USD) per month. Nearly all (99%) reported that there were children and other family members (in most cases their husbands' parents) living in the household.

**Table 1:** Description of study population: spouses of injection drug users enrolled in Madras Injection Drug User and AIDS Cohort Study, Chennai India, 2009 (n = 400)*

	N = 400 (%)
***Median age (years, IQR)***	31 (26 - 36)
***Tamil ethnicity***	388 (97)
***Education***	
None	110 (27.5)
Primary	246 (61.5)
> Primary	44 (11)
***Current marital status***	
Married/living with spouse	318 (79.5)
Married/not living with spouse	28 (7)
Widowed	53 (13.3)
***Median duration of marriage (years, IQR)***	14 (7-19)
***Employment status***	
Unemployed	24 (6)
Weekly/monthly wages	148 (37)
Daily wages	227 (56.8)
***Family income (INR)***	
< 500	258 (66.7)
500-1500	72 (18.6)
>1500	57 (14.7)
***Children living in household***	399 (99.9%)
***Alcohol use***	
Never	298 (74.5)
< 1/week	64 (16)
≥1/week	38 (9.5)
***Ever used non-injection drugs***	88 (22)
Marijuana	6 (1.5)
Smoked/chased brown sugar	7(1.8)
Pharmaceutical drugs	41 (10.3)
Intoxicating tobacco	80 (20)
***Ever injected drugs***	4 (1)
***Lifetime number of sexual partners***	
1	340 (85)
2	52 (13)
>2	7 (2.8)
***Lifetime condom use with regular partner***	
Never	297 (74.3)
Sometimes	75 (18.8)
Always	27 (6.8)
***Ever non-spousal sexual partner***	37 (9.3)
***Condom use with non-regular partner***	
Never	26 (70.3)
Sometimes	7 (18.9)
Always	4 (10.8)
***Ever exchanged sex for money/drugs***	37 (9.3)
***Had sex with someone known to be HIV+***	49 (12.3)

*INR, Indian Rupees

### Risk behavior

Only 4 women (1%) reported a history of injecting drugs. Two of the four had injected in the prior six months. Two reported injecting heroin and the other two reported injecting buprenorphine. All reported injecting with very good friends, but not with their husbands. Twenty-six percent reported some alcohol use but the majority reported drinking < once/week and only 9 women reported daily alcohol use. Among the 102 women who reported any alcohol use, the most commonly consumed beverages were brandy (50%) and a combination of wine and beer (66%). Eighty-eight women (22%) had ever used non-injection drugs. The most common non-injection drug reported was smokeless tobacco (e.g., chewing tobacco products such as mawa, zarda) followed by pharmaceutical drugs.

The majority (85%) reported only one lifetime sexual partner; Fifty-two (13%) reported 2 sexual partners and 7 (3%) reported >2 partners with only 1 reporting more than 4 partners (number of partners = 22) over their lifetime. Thirty-seven (9%) reported having ever exchanged sex for money or drugs. Of the 59 women who reported multiple partners, 37 reported exchange sex and 9 were widowed. Forty-nine (12%) had sex with someone who was known to them to be HIV positive. Condom use with both regular and non-regular partners was infrequent (Table 1). Three-quarters (74%) reported never using condoms with their primary spousal partner and 70% reported never using condoms with their non-regular partners.

### Prevalence of HIV, HCV and HBV

**Figure 1:**
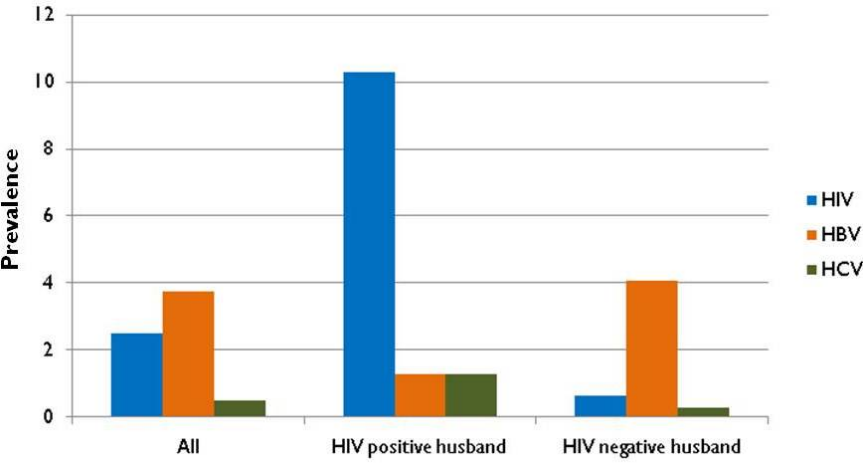
**Prevalence of HIV, hepatitis C virus (HCV) and hepatitis B virus (HBV) among 400 wives of injection drug users by spousal HIV status in Chennai, India (2009)**.

Ten (2.5%) women were positive for HIV antibodies, 2 were positive for HCV antibodies (0.5%) and 15 (3.8%) were positive for HBsAg. Of the 10 women who were HIV positive, 8 (80%) had husbands who were also HIV positive. All 10 reported currently living with their spouse. Three reported two lifetime sexual partners and the remaining 7 reported one. One reported a history of exchange sex. Only three reported always using condoms with their primary partner and none reported always using condoms with their non-regular partners. None reported a history of injection drug use while three reported some alcohol use and two reported some non-injection drug use. HIV prevalence was significantly higher among women whose husbands were also HIV positive (10.2% vs. 0.6%, p < 0.001; Figure 1). Of the 14 who were HBsAg positive, only one had a husband who was also HBsAg positive. Of the two who were HCV positive, one had a husband who was also HCV positive and neither reported a history of injection. Five of the 10 HIV positive women were aware of their status; two women reported a prior positive HIV test result but had a negative antibody result in the study. Overall, 40% had been tested for HIV at least once (Table 2). The majority (94%) had never been tested for HBV or HCV.

**Table 2:** HIV, HBV and HCV testing history among spouses of injection drug users enrolled in the Madras Injection Drug User and AIDS Cohort Study, Chennai, India, 2009 (n = 400)

	N = 400 (%)
***Ever received an HIV test***	122 (40.7)
***Result of last test***	
Negative	87 (71.4)
Positive	7 (5.7)
Do not know/result not received	28 (23)
***Prior testing for hepatitis viruses***	
Not tested for either	375 (94.2)
Tested for one but not sure which	10 (2.5)
Tested for hepatitis B only	1 (0.3)
Tested for hepatitis C only	0
Tested for both	10 (2.5)

Factors significantly associated with HIV prevalence (Table 3) included more stable employment (odds ratio [OR] for weekly/monthly wages vs. daily wages: 5.59; 95% confidence interval [CI]: 1.14, 7.27), higher family income (OR for 500-1500 INR per month vs. < 500 INR per month: 5.0; 95% CI: 1.09, 22.9), always using condoms with regular partner (OR: 9.13; 95% CI: 1.93, 43.2), having had sex with someone known to be HIV positive (OR: 11.6; 95% CI: 3.15, 42.8) and having an HIV positive husband (OR: 17.9; 95% CI: 3.73, 86.3).

**Table 3:** Factors associated with prevalent HIV infection among spouses of injection drug users enrolled in the Madras Injection Drug User and AIDS Cohort Study, Chennai, India, 2009 (n = 400)

	Unadjusted OR (95% CI)
***Age (per 10 years)***	1.05 (0.69 - 1.60)
***Education status***	
None	1
At least primary level education	1.54 (0.32 - 7.35)
***Duration of marriage (per 5 years)***	0.97 (0.63 - 1.50)
***Employment status***	
Daily wages	1
Weekly/monthly wages	5.59 (1.14 - 7.27)
Employed	5.11 (0.45 - 58.7)
***Family income (rupees)***	
< 500	1
500-1500	5 (1.09 - 22.9)
>1500	4.81 (0.95 - 24.5)
***Alcohol use***	
None	1
< 1/week	0.66 (0.08 - 5.42)
≥1/week	2.29 (0.46 - 11.5)
***Ever used non-injection drugs***	0.88 (0.18 - 4.22)
***Lifetime number of sexual partners***	
1	1
>1	2.55 (0.64 - 10.1)
***Lifetime condom use with regular partner***	
Never	1
Sometimes	3.04 (0.67 - 13.9)
Always	9.13 (1.93 - 43.2)
***Ever exchanged sex for money/drugs***	1.06 (0.13 - 8.62)
***Had sex with someone known to be HIV+***	11.6 (3.15 - 42.8)
***HIV status of husband***	
Negative	1
Positive	17.9 (3.73 - 86.3)
***Ever experienced sexual violence***	0.47 (0.12 - 1.85)

*Results from logistic regression analysis; OR, odds ratio; CI; confidence interval

### Partner risk behavior

Three hundred and eight-six (97%) were not aware of their husband's injection drug use prior to marriage. Of these, the majority (91.9%) learned their husbands were IDUs when they saw them injecting at some point during marriage. Eighty-two percent were aware of their husband's HIV serostatus but only 57.9% were aware of their husband's hepatitis status. Women who reported being aware their husband was HIV positive were no less likely to report sexual intercourse with their husband in the prior six months compared to those who perceived their husband to be negative or did not know their status (86.8% vs. 88.5% and 83.6% respectively, p = 0.53). However, among those who were sexually active with their primary partner, those who thought their husband was HIV positive were significantly more likely to always use condoms than those who did not think their husband was HIV positive or did not know their status (15.2% vs. 0.4% and 3.3%, respectively, p < 0.0001). Among those women who thought their husband was HIV positive, 48% reported never using condoms in the prior six months. Women who thought their husbands were HIV positive reported better knowledge regarding protection conferred by condoms against HIV (66% vs 53%, p < 0.01); however, thirty four percent of women who thought their husband was HIV positive did not know that condoms could protect against HIV. Women who thought their husbands were HIV positive were significantly more likely to have had an HIV test previously vs. those who either thought their husband was negative or did not know (64.2% vs. 24.1% and 28.8%, respectively, p < 0.0001). Thirty-six percent of women who were aware of their husbands HIV positive status had never had an HIV test. Forty two percent of women with an HIV positive husband felt that they could not get HIV from their husband if he was not actively injecting; this did not differ from those women who did not have an HIV positive husband (39%, p = 0.9). Other risk behaviors (e.g., exchange sex, alcohol use, non-injection drug use) did not differ by whether their partner was HIV positive.

When women were queried about the potential harmful impact of their husband's injection drug use, the primary fear was that he would get HIV/AIDS (36.7%), followed by the impact on income for the family (33.2%) and being a bad influence on their children (22.4%).

### Intimate partner violence

**Figure 2:**
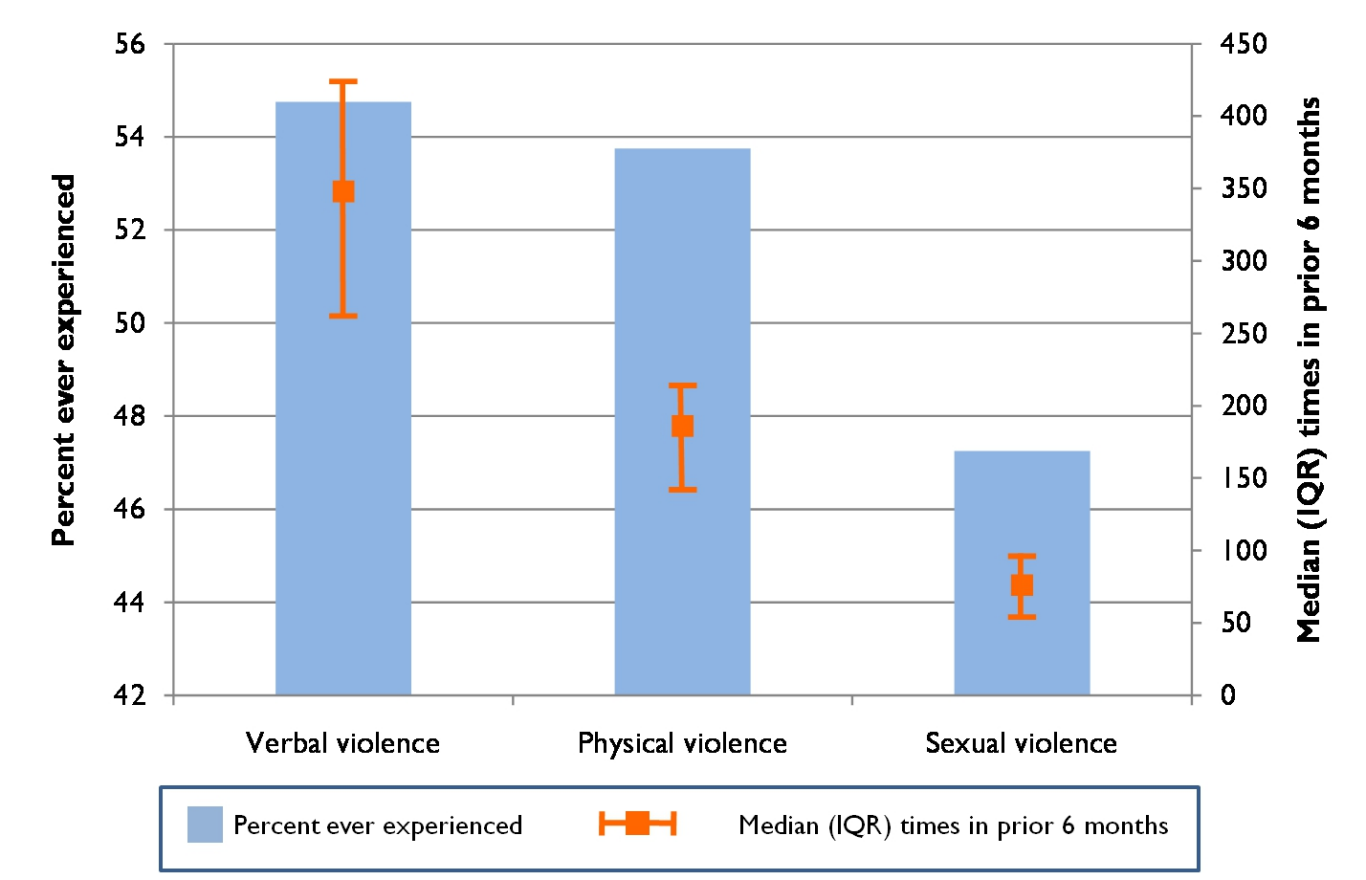
**Prevalence of self-reported experienced intimate partner violence among 400 wives of injection drug users in Chennai, India (2009)**. Bars represent percent ever experienced and lines represent median episodes (interquartile range [IQR]) of experienced violence in the prior 6 months.

Two hundred and twenty-two (55.5%) women reported that they had ever experienced intimate partner violence (IPV). Of these, 127 (56.7%) reported that violence always occurred while their husband was under the influence of alcohol and 90 (40.2%) reported that it sometimes occurred while their husband was under the influence of alcohol. Sixty-nine (30.8%) reported that violence always occurred when their husband was under the influence of drugs and 139 (62.1%) that it sometimes occurred when their husband was under the influence of drugs. Of the women who experienced violence, 98.2% reported verbal violence, 96.4% reported physical violence (beaten/kicked/pushed/slapped by their spouse), 85.5% experienced sexual violence (forced to have sex against their will) and 8.2% had been burned with a cigarette. The overall proportion experiencing different types of violence and the frequency in the prior six months is illustrated in Figure 2. In the prior six months, women experienced verbal violence a median of 348 times (IQR: 262 - 424), physical violence a median of 186 times (IQR: 142-214) and sexual violence a median of 76 times (IQR: 54-96).

## Discussion

HIV prevalence among the spouses of IDUs in this sample in Chennai is about ten times higher than the prevalence of HIV among antenatal clinic attendees in Tamil Nadu (0.25%) [6]. Prevalence of chronic HBV was also high but HCV prevalence was comparable to the expected general population prevalence. The low HCV prevalence despite high prevalence of HIV and HBV confirm that these women have little to no injection-related risk of their own. It appears that the majority of these women are married and monogamous with low risk outside of unprotected sexual intercourse with their husbands. This risk is compounded by the context in which these women appear to be living; women were subject to poverty and high levels of IPV including forced sex particularly when their husbands were under the influence of drugs and/or alcohol. These data further substantiate the importance of including these women in prevention and treatment efforts to reduce bloodborne infection transmission and IPV.

We observed a lower HIV prevalence among the women in this study compared with what has been previously observed, particularly in the Northeast, but our estimate was comparable to prior data from Chennai. Chakrabarti and colleagues reported in 2000 that 45% of the wives of 233 HIV-infected injectors in Manipur were HIV positive [14]; Panda et al reported a prevalence of 5% among spouses of 226 married IDUs from Chennai - 16% of the spouses of HIV-positive IDUs tested positive for HIV antibodies [15]. The higher HIV prevalence among the wives of IDUs in Manipur may reflect an older HIV epidemic in the Northeast as well as a higher prevalence of injection drug use among women in this region compared with others [32]. In both studies, the primary factor associated with HIV aside from HIV/STI status of the partner was duration of HIV infection in the husband [16]. HIV prevalence among these women in Chennai will likely continue to increase particularly given the setting of ongoing sexual violence and low condom use even among women who were aware of their husband's positive HIV status. These data further highlight the need for female-controlled prevention measures.

Our study extends these other studies by including data on HBV and HCV as well as more detailed characterization of risk environment of these women. We observed relatively low HCV prevalence (0.5%) which is consistent with data that suggests that heterosexual transmission of HCV is infrequent [33]. Further, injection was negligible among these women. By contrast, the prevalence of chronic HBV infection was higher. Interestingly, there was little concordance with the HBsAg status of the spouse; however we did not have information on all markers of HBV infection so it is possible that some of their spouses were also infected but had previously cleared (HBsAg negative but anti-HBs positive).

The strongest correlate of HIV was husband's HIV serostatus. Our data are fairly consistent with prior reports from India suggesting that these women have relatively little risk of their own. Less than 1% had ever injected, 85% were monogamous and a very small proportion had more than 2 sexual partners. Though 37 women reported a history of exchanging sex for money or drugs, only one woman reported more than four partners. Not surprisingly condom use was extremely low in this population reflecting general trends among married couples in India [18-20]. The positive association between condom use and HIV infection likely reflects higher levels of condom use among those who were aware of their HIV status as has been previously observed in India and other settings [20,34]. Yet, it is important to note that even among the women who were aware that their husbands were HIV positive, consistent condom use was not universal and rates of HIV testing were low.

Prior studies from India and South Asia have also suggested low rates of condom use among similar groups of women [16,17,35] and low testing rates [15,17]; however few of these studies had information to link behavior with women's knowledge of their husband's serostatus. Therefore, it is particularly concerning that such behaviors were low even among those women who believed their husband was infected. Future studies should explore in more depth, possibly qualitatively, the reasons for these low rates. Our data suggest that knowledge plays a role given that 33% of women with an HIV positive spouse did not know that condoms could protect against HIV and 42% did not know they were at risk for HIV if their husband was not actively injecting. Beyond knowledge, the reasons for low condom use are likely multifactorial and include fear [36], the idea that unprotected sex in long-term partnerships is a sign of intimacy and a request for condom use a sign of distrust [17] and that the power and control in sexual relationships in this region is unequally distributed by gender [37]. It is not uncommon for women in south India (especially from low income groups) to be subject to verbal/physical violence for suggesting condom use during sexual intercourse with a primary partner [38]. As has been done in other settings, interventions targeting IDUs need to focus on issues beyond risks associated with needle sharing and need to target their sexual partners. Drop-in centers and voluntary counseling and testing (VCT) centers targeting IDUs should include services for sexual partners (VCT, condom distribution, targeted information, education and communication (IEC) sessions), as has been recommended by the World Health Organization, United Nations Office of Drugs and Crime (UNODC) and UNAIDS as a component of the comprehensive package of services for IDUs [39].

Second to HIV/AIDS, the most pressing concern for these women was the impact their husband's drug use had on income of the family. The median income reported by the married IDUs enrolled in MIDACS was ~1500 INR per month compared to a median 500 INR reported by the spouses suggesting that IDUs might have been using a large proportion of their income for drugs/alcohol. The positive association between stable employment and HIV may be further reflective of the strain placed on these families. Husbands who are riskier (e.g., inject more frequently) may consequently bring less income home forcing their wives to seek stable employment. Further, it is also possible that women seek stable employment after their husbands become ill with complications of HIV or die as has been seen before in India [20]. Indeed 70% of HIV positive women worked for weekly or monthly wages vs. 37% of HIV negative women. Further, 13% of the spouses of the women enrolled in this study died within three years of enrolling in the MIDACS. It is possible then that at least some of these women were forced to work to support their families.

The economic adversity is further exacerbated by a climate of violence and potentially fear. We observed high levels of intimate partner violence, both physical and sexual in this population. Sexual and physical violence have been previously reported to be common in India [38]. While direct comparisons of prevalence are difficult due to differences in definitions of physical and sexual violence, the lifetime prevalence of sexual violence of 50% appears to be higher than other studies, even those conducted in Chennai with other high risk groups (e.g., men in wine shops) [25,26,28]. This likely reflects the added effects of substance abuse in a population where baseline levels of sexual violence are already higher than normal. Even more alarming than the lifetime prevalence of violence were the frequency of recent episodes of both sexual and physical violence suggesting that on average, violence occurs daily. Sexual violence was not associated with higher HIV prevalence but we were not able to perform multivariate analysis to rule out negative confounding. Further, the lack of association may simply reflect the overall high prevalence of sexual violence.

India is a strong patriarchal society and specific gender roles are embedded deep within the culture. A key role of men is to provide financially for their families. When men are injecting drugs, this impacts their ability to function in this role causing tremendous strain on their wives and families. Women are somewhat powerless in this situation as the majority of these women marry without knowing about their husband's drug use. When they ultimately find out, it is too late for them to change their situation as divorce is not commonplace in Indian society, particularly in lower socioeconomic communities. This situation appears to produce a volatile environment, one in which resources are strained and stability is thereby affected, leading to high levels of violence and disease transmission.

We were limited in this analysis by self-reported data, which may be subject to social desirability, particularly in this population where high-risk behavior is not normative. However, all participants were reassured regarding confidentiality and all interviewers were women to ensure the highest degree of comfort possible. Also, the women were reassured that none of the information shared with the interviewers would be relayed to their husbands. Due to the cross-sectional nature of the study, we were not able to establish temporal associations between exposures and HIV and other outcomes. Indeed, some of the associations observed (e.g., HIV and condom use) may reflect reverse causation. Further, the small number of HIV infections (n = 10) prevented us from conducting multivariate analysis. It is possible that some of the associations we observed or did not observe would have changed after accounting for confounding.

## Conclusion

These data reinforce that HIV epidemics do not end with the persons engaging in high-risk behaviors. Spouses of IDUs represent another group of mostly monogamous women in India who are placed at risk for HIV by the behavior of their partners. Their risk context is further strained by compromised economic resources and high levels of intimate partner violence. Our data coupled with other investigations support that HIV prevention and treatment programs targeted at IDUs should also include components directed at their families. Strategies to empower these women are clearly needed; of particular interest may be interventions that provide economic opportunities for these women. Trials of pre-exposure prophylaxis (PrEP) and female-controlled prevention tools such as vaginal microbicides should attempt to recruit this population at high-risk for HIV acquisition. In the meantime, while such novel tools await efficacy demonstration, all programs providing services to IDUs need to include services targeting wives/sexual partners of IDUs (VCT, condom distribution, couples counseling, etc.) as recommended by the WHO, UNAIDS and UNODC.

## List of Abbreviations

CI: Confidence interval; FSW: female sex worker; HBV: hepatitis B virus; HCV: hepatitis C virus; HIV: human immunodeficiency virus; INR: indian rupee; IDU: injection drug use; IQR: interquartile range; IPV: intimate partner violence; MIDACS: Madras Injection Drug User and AIDS Cohort Study; MSM: men who have sex with men; OR: odds ratio; STI: sexually transmitted infection; USD: United States dollar; VCT: voluntary counselling and testing; YRGCARE: YR Gaitonde Centre for AIDS Research and Education; YRGCSAR: YR Gaitonde Centre for Substance Abuse Research

## Competing interests

The authors declare that they have no competing interests.

## Authors' contributions

SSS conceived the study, participated in its design, conducted the analysis and helped to draft the manuscript. AKS participated in the design and supervised data collection. DDC participated in the design. SCJ participated in qualitative and quantitative data collection. KGM carried out laboratory testing. SA participated in data management and analysis. MSK participated in the design and critical review of manuscript. SS participated in the design and critical review. SHM conceived the study, participated in design and drafting of manuscript. All authors read and approved the final version of the manuscript.

## Pre-publication history

The pre-publication history for this paper can be accessed here:

http://www.biomedcentral.com/1471-2458/11/39/prepub

## Supplementary Material

Additional file 1 **Risk assessment questionnaire**. This is the behavioral survey that was administered to the 400 women in this study Click here for file
