# Supplementary material for: The intersection between sex and drugs: a cross-sectional study among the spouses of injection drug users in Chennai, India
Source: BMC Public Health. 2011 Jan 16;11:39. doi: 10.1186/1471-2458-11-39 (PMC3031221; doi:10.1186/1471-2458-11-39)
Supplement: Additional file 1 — Risk assessment questionnaire. This is the behavioral survey that was administered to the 400 women in this study [file 1471-2458-11-39-S1.DOC]

**MIDACS II RISK BEHAVIOR QUESTIONNAIRE**

**Participant eligibility / introduction**

**Participant ID**

**YES NO**

**YES NO**

**Inclusion**

1. Is the participant of legal age to provide written informed consent for research?

2. Did the participant provide written informed consent for screening and study participation? ...........................................................................................................

dd MMM yy

3. Interview date:

**Exclusion**

4. Does the participant have any obvious psychological disturbance or cognitive impairments that would limit his or her ability to understand study procedures? ………………………………………………………………………..

(as determined by clinic staff)? ...............................................................................

5. Does the participant have, in the opinion of the interviewer, any other condition that would make participation in this study unsafe or otherwise interfere with study objectives? ..............................................................................................................

**If no to any, participant is ineligible.**

**If yes to any, participant is ineligible.**

4. Interviewer initials

**Read to the participant :**

We are going to ask you some questions to better understand who you are. Some of the questions may be personal. When I read each question, if anything is unclear, please stop me and I will try to make the question clearer.

Of course, your responses will be confidential. Your name will not be reported to anyone nor will any of the information be connected with you because an identification number is being used instead of your name. If you cannot or do not wish to answer a particular question, tell me and I will go on to the next question. Please remember that there are no right or wrong answers. Just answer as best and as truthfully as you can.

**Participant ID**

# Demographics

1. What is your date of birth?

**OR** Age: years (estimate OK)

Male female

dd MMM yy

2. What is your sex? .......................................................................................

3. What is your Ethnicity?

Tamilian

Telangana

Malayalee

Kannadiga

other, **specify**:

Married / living with spouse None

4. What is your current marital/living status? 4a. What is your highest level of education?

Married / not living with spouse Primary Schooling

Not married / living with partner Secondary school

**Skip to question 6**

Not married / not living with partner High school

Widowed Vocational or trade school

Completed University

5. For how many years have you been married?

years

Graduate/Professional

6. Are you currently a student? ...........................................................

Yes no

7. Which best describes your current employment?

8. Average monthly family earnings in Rupees

Not employed / homemaker

9. Who currently lives in your household? **(Select all that apply)**

Monthly Wages

Weekly Wages

Daily Wages

Less Than 500

500 - 1500

1501 - 3000

Above 3000

Husband Children Parents Other family members Other, **specify**:

________________________

# HIV/AIDS Knowledge

**Read to the participant :**

We are now going to ask you some questions to assess your knowledge of HIV and AIDS. There are no correct or wrong answers. Please remember that you can refuse to answer any of the questions if they make you uncomfortable. However, try to be as truthful as you can while answering the questions, as your answers to these questions are important for the research. Also remember that the information that you provide here will be highly confidential.

**DON’T**

**YES NO KNOW**

10a. There is a cure for AIDS. .............................................................................................

10b. Coughing and sneezing can spread HIV…………………………………………………

10c. HIV can be spread by mosquitoes…………………………………………………………

10d. A person can get HIV by sharing a glass of water with someone who has HIV……….

10e. A pregnant woman with HIV can give the virus to her unborn baby…………………….

10f. A woman can get HIV only if she has vaginal sex with a man……………………………

10g. A woman cannot get HIV if she has anal sex with a man………………………………...

10h. Showering or washing one’s genitals after sex keeps a person from getting HIV……..

10i. All pregnant women infected with HIV will have babies born with HIV/AIDS…..……….

10j. Using a condom can lower a person’s chance of getting HIV……………………………

10k. A person with HIV can look and feel healthy………………………………………………

10l. Some drugs are available for treatment of HIV……………………………………………..

10m. A person can get HIV even if she/he has sex with another person only one time……

10n. A woman cannot get HIV if she has sex during her period………………………………

10o. You can tell someone has HIV by looking at them……………………………………….

10p. Having sex with more than one partner increases ones chances of getting HIV………

10q. A person can get HIV through contact with saliva, tears, sweat or urine………………

10r. Washing drug use equipment/needles with cold water kills HIV…………………………

10s. Eating healthy foods prevents you from getting HIV………………………………………

10t. An injection drug user can get HIV by sharing needles with someone who is HIV infected……………………………………………………………………………………………….

10u. A spouse/sexual partner of an IDU can get HIV from their husband/sexual partner who is an IDU even if the spouse/sexual partner is not injecting………………………………

10v. A spouse/sexual partner of an IDU can get Hepatitis B or C from their husband/ partner who is an IDU even if the spouse/sexual partner is not injecting…………………. . ..

**Participant ID**

# Drug and Alcohol use

**YES NO**

**YES NO**

**YES NO**

**YES NO**

**YES NO**

**Read to participant:** The next few questions are about alcohol use.

12. Using this card, tell me, on average, how often do you have a drink? Show Card #1. (Check only one)

# of drinks

**ALCOHOL USE**

less than once per week

1–2 days 3–4 days 5–6 days per week per week per week

Every Day

less than once per month

11. Have you ever consumed alcohol?

13. On days that you drink, how many drinks do you usually have?...................................... ………...........................

# of drinks

14. On the days that you drink, what do you drink? (**Check all that apply**)

Whisky

Rum

Brandy

Vodka

Red Wine and Beer

Beer

Other, **specify** :**_**____________________________________________________

**Read to participant:** The next few questions are about drug use.

**DRUG USE**

15. Have you **EVER** done any of the following…?

15a. smoke ganja? ......................................................................................

15b. smoke brown sugar?...........................................................................

15c. chase brown sugar? ………………………………………………..

15d. buy and use any medicine from any pharmacy for non medicinal use?(Sleeping tablets, Avil, Calmpose, Restyl)…………………..

15f. use any other drug? e.g. Abin, bhang, amphetamines (specify)……….

_________________________________

15e. chew mawa / zarda / any other form of intoxicating tobacco? ………...

**If no, skip to question 15**

**Participant ID**

**YES NO**

16. Have you **EVER** used a needle to inject any drugs under your

skin or into a vein? ..............................................................................................

**If no, skip to question 19**

16a. Did you do this in the **last six months**? .................................................

17. Which of the following substances have you **EVER** injected?

17a. heroin (brown sugar)? …………………………………………………….. … ………………………............................................................................................

**YES NO**

17b. Buprenorphine (Tidigesic)? ……………………………………………........

17c. Avil? ………………….................................................................................

17d. Diazepam (Calmpose)?.……………………………………………………….

17e. Promethazine (Phenargan)? ….………………………………………….......

17f. Others (Celin,etc) Specify ____________________________________

17g. the above drugs in any combination? ………………………………………..

18. When you injected, who did you inject with?

Spouse/primary sexual partner

Non-primary sexual partner

Very good friend

Acquaintance (friend you do not know well)

Dealer

Other, **specify**:_____________________________________

**YES NO**

**YES NO**

**Participant ID**

**If 0, go to Q21b**

**Never Sometimes Always**

**YES NO Refused**

**YES NO Refused**

**Never Sometimes Always**

**Never Sometimes Always**

**YES NO Don’t know**

20. In general how often have you used a condom with your regular sex partner?.. ............................

19. In your lifetime, how many men have you had vaginal or anal sex with?........

21. In the **last six months**, how many times did you have vaginal or anal sex with your primary sex partner? ......................................................

21a. How many of these times did you (or your partner) use a condom? ......

24. In the **last six months**, how many times did you have vaginal or anal sex with someone other than a primary sex partner? .......................................................

24a. How many of these times did you use a condom? .........

25. Have you ever exchanged sex for money or drugs?...........................................

27. In the **last one month**, how many sex partners gave you money or drugs in exchange for sex?. ..........................................................................................

21b. Did you use a condom during your last sexual encounter with your regular partner?............................................................................................

24b. Did you use a condom during your last sexual encounter with a non-regular partner?............................................................................................

**Read to participant:** The next few questions are about sexual behavior. These are very personal questions. Please remember that all of your responses are confidential.

22. In your lifetime, have you ever had vaginal or anal sex with someone other than your spouse/primary sexual partner?................................................................

**If no or refused, go to Q25**

**If no or refused, go to Q28**

23. In general how often have you used a condom with your non- regular sex partner(s)?.. ..............................................................................................................

26. In general when you exchanged sex for money or drugs, how often did you use a condom?.. .......................................................................................................

28. Have you ever had vaginal or anal sex with someone you knew was HIV positive?..................................................................................................................

# Sexual risk behavior

**If 0, go to Q24b.**

**Positive Negative Don’t know Result not received**

**Participant ID**

**HIV / hepatitis testing history**

**Yes No Don’t know**

**Read to participant:** We are now going to ask you some questions about your husband / primary sexual partner. Some of these questions may be personal and you can refuse to answer any of them. There are no right or wrong answers and your answers will be kept confidential.

29. Have you ever been tested for HIV?................................................................

**If no or don’t know, go to Q31**

30. What was the result of your last HIV test?.............

31. Have you ever been tested for hepatitis (hepatitis B or hepatitis C)?

Tested for both

Tested for hepatitis B

Tested for hepatitis C

Tested for hepatitis but do not know if it was B or C

Not tested for either

Do not know

Results not received

32. What were the results of your last hepatitis tests?

Positive for both hepatitis B and C

Positive for hepatitis B only

Positive for hepatitis C only

Positive for hepatitis but do not know if it was B or C

Negative for both

Negative for hepatitis but do not know if it was B or C

Do not know results of either test

33. Considering all of the behaviors that could have put you at risk for HIV, how would you rate your overall risk for HIV in your lifetime?

Not risky at all

A little risky

Somewhat risky

Mostly risky

Very risky

**Skip to question 33**

**Partner risk behavior**

**Participant ID**

**Read to participant:** We are now going to ask you some questions about your husband / primary sexual partner. Some of these questions may be personal and you can refuse to answer any of them. There are no right or wrong answers and your answers will be kept confidential.

**Yes No Don’t know**

**Yes No**

**Yes No Don’t know**

34. When did you first learn that your spouse/sexual partner was an IDU?

Before marriage (before dating if not married)

After marriage (after dating if not married)

35. How did you first learn that your spouse/sexual partner was an IDU?

Saw him injecting / with paraphernalia

Family member told you

He told you himself

Someone else told you

He was high and you recognized this

36. Does your spouse/sexual partner have HIV?..................................................

37. When was your spouse/sexual partner diagnosed?

38. Have you seen the report that says he/she does/does not have HIV?.............

year of diagnosis **OR** years ago

**If no or don’t know, go to Q38**

39. Does your spouse/sexual partner have hepatitis?...........................................

**If no or don’t know, go to Q41**

40. When was your spouse/sexual partner diagnosed?

year of diagnosis **OR** years ago

41. Which of the following things concern you about your spouse/sexual partner’s injection drug use behavior?

**(Select all that apply)**

Affects income generation for the family

Fear he/she will get HIV/AIDS

Fear you will get HIV/AIDS

Bad influence on your children

Fear of violence towards you

Other (please specify:___________________________________________________________________

**Participant ID**

**Yes No Refused**

**Never Sometimes Always**

**Never Sometimes Always**

42. Of the concerns you listed above, what is your **PRIMARY** concern about your husband’s injection drug use behavior? **(Select only one)**

Affects income generation for the family

Fear he will get HIV/AIDS

Fear you will get HIV/AIDS

Bad influence on your children

Fear of violence towards you

Other (please specify:___________________________________________________________________

43. Has your spouse/sexual partner ever been violent towards you?....................

44. Does this occur when he is under the influence of alcohol?............................

45. Does this occur when he is under the influence of drugs?..............................

**If no or refused, go to Q48**

46. Which types of violence by your spouse/sexual partner have you experienced? (**Select all that apply**)

Verbal violence (screamed at you)

Physical violence (beat you)

Burned you with a cigarette

Forced you to have sex against your will

Other, **specify**:___________________________________________________________________

Refused

**If refused, go to Q48**

47. In the **past 6 months**, how many times have you experienced each of the following types of violence by your spouse sexual partner? (**Read only those selected in Q46**)

Verbal violence (screamed at you)………………………

Physical violence (beat you)……………………………...

Burned you with a cigarette………………………………

Forced you to have sex against your will ……………….

Other………………………………………………………...

**Yes No Don’t know**

**Yes No Don’t know**

48. Is your spouse/sexual partner currently injecting?.....................................

**If no or don’t know, go to Q50**

49. Has your spouse/sexual partner ever tried to stop injecting drugs?.........

**If no or don’t know, go to Q51**

50. What were some of the factors that were influential in getting him/her to stop?

You or someone else in your family asked him to stop

Fear of HIV/AIDS

Lack of money

Drugs were no longer available

He went to jail

Relocation

Drug treatment

Other (please specify:___________________________________________________________________

51. What are the factors that you think would most help your spouse/sexual partner stay away from injection drugs? **(Select all that apply)**

Drug treatment (sublingual, detoxification)

Having a proper job/source of income

Counseling

Detoxification

Relocation to a drug-free neighborhood

Other (please specify:___________________________________________________________________

52. Of the factors that you listed, which do you think is the most important factor that would help your spouse/sexual partner stay away from injection drugs? **(Select only one)**

Drug treatment (sublingual, detoxification)

Having a proper job/source of income

Counseling

Detoxification

Relocation to a drug-free neighborhood

Other (please specify:___________________________________________________________________
